# What's the Cost? Measuring the Economic Impact of Pediatric Sepsis

**DOI:** 10.3389/fped.2021.761994

**Published:** 2021-11-15

**Authors:** Erin F. Carlton, Scott L. Weiss, Hallie C. Prescott, Lisa A. Prosser

**Affiliations:** ^1^Division of Critical Care Medicine, Department of Pediatrics, University of Michigan, Ann Arbor, MI, United States; ^2^Susan B. Meister Child Health Evaluation and Research Center, Department of Pediatrics, University of Michigan, Ann Arbor, MI, United States; ^3^Department of Anesthesiology and Critical Care, Children's Hospital of Philadelphia, University of Pennsylvania Perelman School of Medicine, Philadelphia, PA, United States; ^4^Pediatric Sepsis Program, Children's Hospital of Philadelphia, Philadelphia, PA, United States; ^5^Veterans Affairs Center for Clinical Management Research, Health Services Research & Development Center of Innovation, Ann Arbor, MI, United States; ^6^Department of Internal Medicine, Division of Pulmonary and Critical Care, University of Michigan, Ann Arbor, MI, United States; ^7^Department of Health Management and Policy, School of Public Health, University of Michigan, Ann Arbor, MI, United States

**Keywords:** sepsis, family, finances, pediatric, financial toxicity

## Abstract

Sepsis, life-threatening organ dysfunction secondary to infection, hospitalizes nearly 75,000 children each year in the United States. Most children survive sepsis. However, there is increasing recognition of the longer-term consequences of pediatric sepsis hospitalization on both the child and their family, including medical, psychosocial, and financial impacts. Here, we describe family spillover effects (the impact of illness on caregivers) of pediatric sepsis, why measurement of family spillover effects is important, and the ways in which family spillover effects can be measured.

## Introduction

Sepsis and septic shock result in the hospitalization of nearly 75,000 children each year in the United States (1). Increasingly, the impacts of sepsis beyond acute hospitalization are recognized. Children commonly experience declines in quality of life (QOL) (2–4), increased need for subspecialty care (5), and new dependence on medical devices (6). It is clear that sepsis causes long-term sequelae in children. What is less known, however, is the impact of sepsis on a child's family.

Post-Intensive Care Syndrome Pediatrics (PICS-p) is a framework which describes the broad sequelae of critical illness, such as sepsis, that are experienced by children and their families (7, 8). Importantly, PICS-p recognizes the bidirectional relationship of a child and their family, with childhood illness affecting caregiver health, and caregiver health subsequently influencing a child's recovery from illness (9). Caregivers often experience stress, anxiety, and depression during and after their child's sepsis hospitalization (10) which can impair a child's recovery.

Beyond exerting a toll on caregiver physical and mental health, sepsis hospitalization may also result in financial costs (e.g., co-pays, transportation, lodging) and additional caregiving burden (e.g., remaining at a child's bedside during hospitalization, a child's new medical needs, follow-up appointments). This financial and caregiving burden can in turn influence recovery. For example, a child's sepsis hospitalization may limit caregivers' ability to work outside the home, thereby causing or exacerbating financial strain, which may impair a child's return to health. The effect of a patient's health on family outcomes, termed “family spillover effects,” has been studied extensively by economists in chronic illnesses (11) such as Alzheimer's Disease (12), autism (13–15), and childhood cancer (16). In this article, we describe family spillover effects, why they should be evaluated in pediatric sepsis, and how they can be measured.

## What Are Family Spillover Effects?

**Figure 1:**
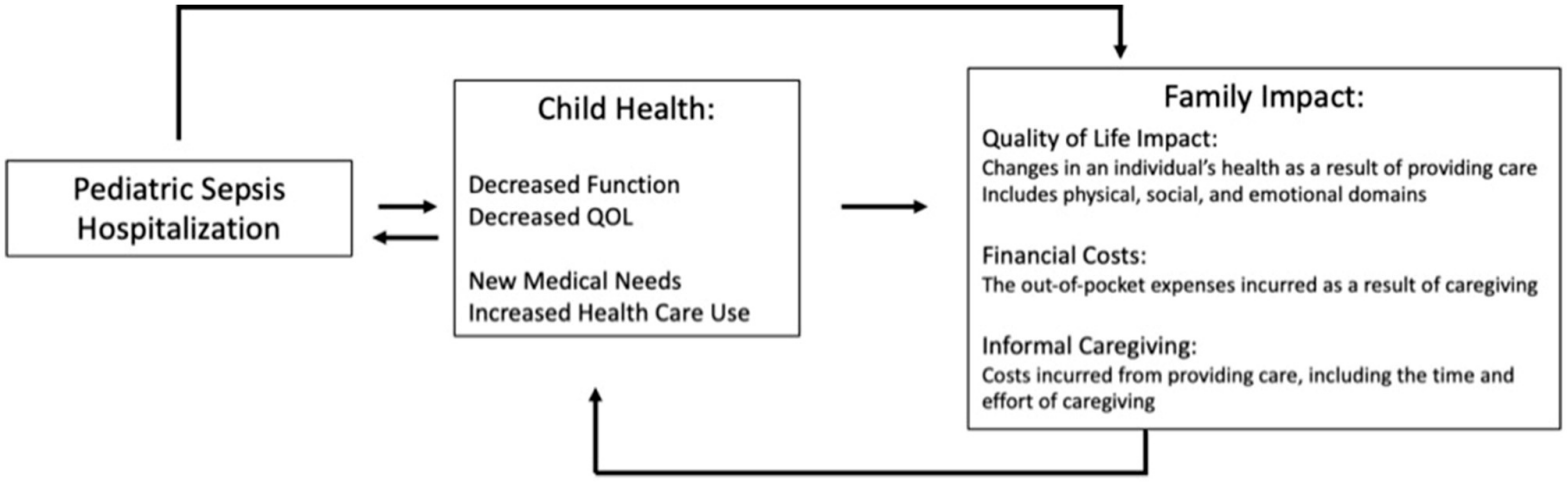
Conceptual model of family spillover effect in pediatric sepsis. Sepsis impacts a child's health in multiple domains including medical, functional, and quality of life, which in turn impacts their family. The family spillover effect encompasses three domains; Quality of Life; Financial Costs, and Informal Caregiving. Quality of life is often measured through survey or scale questionnaires regarding an individual's health in which a numerical value can be attached allowing for comparisons to other groups and economic evaluations including cost-effectiveness analyses. Examples include the CarerQoL, EQ-5D, Health Utilities Index. Financial costs can be measured via prospective or retrospective surveys recalling out-of-pocket expenses, which include both medical (e.g., supplies) and non-medical expenditures (e.g., home remodel) or via administrative databases which provide co-pays or deductibles paid as a result of a child's illness. Informal caregiving can be quantified via diary (recording all of one's activities over a period of time) or recall (recording how much time is spent on a given activity during a period of time).

First described in 2005, family spillover effects recognize that the change in an individual's health can have substantial impacts on the QOL of their family members (17). Simply put, family spillover effects are the direct and indirect impacts of a patient's health on the financial, emotional, and physical health of their family members or caregivers. These effects can be negative (e.g., financial strain, poor sleep) or positive (e.g., sense of fulfillment) (18). Spillover effects are often organized into three categories: QOL impacts, financial costs, and informal caregiving costs (Figure 1). QOL impacts describe the physical, emotional, and social impact resulting from having a sick loved one. The financial costs include out-of-pocket costs such as co-pays, deductibles, and other costs of care not covered by insurance (e.g., transportation, durable medical equipment). Informal caregiving costs encompass the unreimbursed care provided by family members (19), and includes the time-cost involved in caregiving (20).

Family spillover effects result both from providing care and from worrying about others (12, 21). Those affected are not limited to the nuclear family or those providing care, and may include siblings, friends, or other relatives (12). Similarly, spillover effects involve physical and emotional health and well-being, interpersonal relationships, and finances or employment. Spillover effects may subsequently cause the patient to have feelings of guilt or worry. For example, a child recovering from sepsis may feel guilt about their parent's stress or work absence.

While spillover effects have been studied primarily in the context of chronic disease, acute illness and hospitalization likewise impact caregivers QOL and result in both financial and informal caregiving costs. Parents or caregivers report decreased QOL, especially during the acute hospitalization period (22, 23). Families of hospitalized children commonly describe significant financial costs (24), most often related to their child's medical care. Similarly, a child's health impacts parental employment, with higher rates of unemployment or leaving the workforce among family members of children with a chronic illness (25, 26). The need for informal caregiving may result in frustration for employed parents as they describe a lack of leave entitlement and ability for flexible arrangements needed for child care (27). While the Family Medical Leave Act provides eligible workers up to 12 weeks of leave, only 56% of U.S. employees are eligible and the leave is unpaid, which may further exacerbate these family spillover effects (28). Indeed, 40% of parents of children with special health care needs report returning to work sooner than what the parent thought was needed for their child's health, and this was largely due to financial need (29). As children with chronic illness account for a significant portion of pediatric sepsis hospitalizations (30), it is likely many families are experiencing these challenges during hospitalization and beyond.

### Evidence of Spillover Effects of Sepsis

Pediatric critical illness, such as sepsis or septic shock, greatly impacts parent and caregiver mental health and health-related QOL(HRQOL) (Figure 1). Many parents experience acute stress disorder, post-traumatic stress symptoms, anxiety, or depression (8). Children with septic shock can experience emotional difficulties which in turn lower their HRQOL (31). Among adult patients with chronic critical illness or sepsis, caregivers often experience depression, post-traumatic stress symptoms and decreased HRQOL following their loved one's ICU hospitalization (32–34).

After pediatric sepsis, many children experience declines in health, which may increase family financial burden and need for informal caregiving. For example, 1 in 20 children have a new medical device placed during their sepsis hospitalization (6). Tracheostomy for prolonged mechanical ventilation, one of the most common procedures performed during sepsis hospitalizations, has been associated with significant hardship for families (35). Additionally, there is a substantial increase in the number of outpatient appointments in the year after sepsis hospitalization, reflecting the increase in medical complexity and/or vulnerability after sepsis (5). Finally, nearly one-third of children with sepsis are re-hospitalized in the months following discharge (36). Taken together, the high burden of care and risk for further health deterioration after pediatric sepsis may result in several spillover effects including parental stress, increased caregiving requirements, work absenteeism, and financial burden.

## Why Should We Measure Family Spillover Effects in Sepsis?

Family spillover effects can encompass a wide range of individuals and domains. These family spillover effects must be measured in order to quantify the full burden experienced by a family unit (12). Furthermore, recognizing the impact of sepsis on families may improve the understanding and care of pediatric sepsis survivors—for example, by identifying when family financial strain limits the ability to follow-up or carry out the medical plan (18). Recognition of these spillover effects is important not only during sepsis hospitalization, but also after hospitalization when the majority of financial costs and informal caregiving costs occur (37). Indeed, the importance of family is highlighted within the PICS-p framework (7, 8), with calls to action for further study and understanding (9).

In order to deliver the most effective and efficient health care, we must not only look at patient centered outcomes, but also understand the consequences of both the acute illness and interventions rendered (38). To do this, especially within pediatrics, the health, social, and economical effects on families must be studied. Indeed, in addition to their child's health and well-being, families prioritize the outcomes of overall family well-being, social and financial support after critical illness (39).

The formal measurement of family spillover effects differs from the assessment of family outcomes in PICS-p since measurement of spillover effects quantifies the specific economic burden or cost of disease. Because of this formal quantification, family spillover effects can be incorporated into cost-effectiveness and cost-utility analyses to directly measure the relative value of specific interventions. And while the negative health impacts of pediatric sepsis should in itself be enough to drive policy change such as paid time off, finite resources require funders to examine not only health benefits, but the resources required (38). Economic evaluations provide clinicians and policy makers alike a more complete assessment of the value of an intervention (the added benefit and at what cost) (40). This can be applied when comparing interventions in a clinical setting, as well as when considering allocation of financial and resource support (40). A recent systematic review of economic evaluations of adult sepsis demonstrated a large gap in the literature, with many commonly used inventions, such as vasoactive medications and steroids, not included in prior economic evaluation studies (41). Quantifying the impact on the family and measuring the specific economic cost of interventions may drive policy geared toward supporting families and children such as in-home nursing care, transportation/parking stipends, or reimbursement for caregiving.

In the past decade, post-discharge outcomes have been increasingly measured in children hospitalized for critical illness (42). Nearly 20% of studies included in a recent scoping review of outcomes after pediatric critical illness included evaluation of family members (42). Measures of family outcomes largely focus on HRQOL and mental health, such as anxiety, depression, and post-traumatic stress—but the financial costs and caregiving costs are rarely measured.

## How Can we Measure Family Spillover Effects in Sepsis?

The three domains of family spillover effects (QOL, financial costs, and caregiving costs) are most often measured through survey or scale methodologies. Qualitative studies have also described the financial impact of critical care hospitalization on family members and caregivers (43), but quantitative enumeration of family spillover effects is needed for inclusion in an economic evaluation. Finally, spillover effects may be measured via linkage to other administrative data sources, such as employment records.

Quantitative surveys of family spillover effects vary in subject domain, length, and target population and largely focus on QOL measures. Commonly used surveys include the Medical Outcomes Study 36-item Short-Form Health Survey, PedsQL (measure of child QOL), CarerQoL, EQ5D, Self-rated Scale for Post-Traumatic Stress Disorder, General Health Questionnaire, and Parental Stressor Scale: Pediatric Intensive Care Unit (Table 1). They have been used in a general ICU (42, 51, 52), pediatric chronic conditions (53–55), and sepsis (2–4, 56) populations. Some of these measures, including the EQ5D, can be used to derive Quality Adjusted Life Years (QALYs) and support cost-effectiveness analyses (57). Additionally, QOL can also be assessed through direct valuation techniques such as standard gamble, in which participants are asked to choose between two options, one that is certain and one that is not, or time-trade off which asks participants to choose between time at a certain health state versus shorter time in full health, followed by a lesser health state (58, 59). Finally, surveys such as the Comprehensive Score of Financial Toxicity (48), InCHARGE Financial Distress/Financial Well-being Scale (49), and Financial Distress Questionnaire (50) have been used to capture the financial burden of illness among families.

**Table 1:** Select instruments used to measure family spillover effects.

**Instrument**	**Description**	**Number of questions**	**Information source**	**Availability**	**Adapt to quality adjusted life years**
**Quality of life**					
Health utility index (44)	Measures health status and health related QOL including dimensions: vision, hearing, speech, sensation, fertility mobility, pain, dexterity, self-care, emotion, and cognition	15(Self-administered) 40(Interviewer administered)	Self; Proxy	Proprietary	Yes
36-Item short form survey (45)	Survey which assesses eight health concepts including limitations in physical, social, or usual role activities, pain, mental health, energy, fatigue, and general health perceptions	36	Self	Proprietary	No
EQ-5D (46)	Measures five dimensions of health including mobility, self-care, usual activities, pain/discomfort, anxiety/depression	5	Self/Proxy	Proprietary	Yes
CarerQOL (47)	Assesses seven dimensions of caregiving burden including fulfillment, relationship problems, mental health problems, problems with daily activities, financial problems, support, and physical problems	8	Self	Non-proprietary	Yes
**Financial toxicity**					
Comprehensive score of financial toxicity (48)	Measures the financial distress experienced by cancer patients	11	Self	Proprietary	N/A
InCHARGE Financial distress/financial well-being scale (49)	Survey designed to measure the level of well-being and stress due to an individual's personal financial state	8	Self	Proprietary	N/A
Financial distress questionnaire (50)	Designed to assess a person's financial ability to afford everyday items	2	Self	Proprietary	N/A

*The instruments listed in this table provide examples of tools used to in economic evaluations to measure family spillover effects. This is a non-exhaustive list. N/A, Not Applicable*.

While economic burden has been measured by linking national survey studies to administrative claims data among adult patients (60), this has been less readily available for many pediatric populations. Thus, primary data collection, such as surveys or family diaries to measure financial burden, are often needed. This methodology relies on survey and applied economic techniques and has been used in other pediatric populations (61). The economic burden following sepsis or other critical illness may be represented by out-of-pocket costs, work absenteeism, or change of employment, as family members typically provide substantial informal caregiving following a critical care hospitalization. Primary data collection on these measures and establishing a repository of data on family spillover effects will allow for a more complete measurement of the economic burden (41).

## A Call to Action

Post-hospitalization and long-term outcomes have become a significant focus and priority of pediatric critical care and sepsis research. As laid out in the PICS-P framework, the physical, cognitive, emotional, and social health toll of critical illness should be measured bearing in mind the interdependent relationship of family and child (7). In order to effectively do so, consideration and inclusion of the economic and financial impact is required. Indeed, this was recognized as a priority to improve sepsis survivorship (62).

Providing support to families and children impacted by sepsis can take many forms. As families indicate a flexibility in work hours and location as fundamental to maintain a “work-life” balance (27), employers should be more accommodating in their support, perhaps applying the lessons-learned of remote work during the COVD-19 pandemic. Alternatively, respite care programs have been shown to be beneficial for the overall well-being of parents and caregivers (63, 64). In some instances, family members can be employed as home health care aides. However, such programs are not available in all states and often do not permit parent/guardian participation, thus the American Academy of Pediatrics has recommended expansion of these programs and inclusion of parents (65). Finally, financial support programs may provide benefit to caregivers (66). Grants and other patient assistance programs can help with out-of-pocket costs. However, these are often disease or medication specific, limiting their generalizability.

Financial burden and informal caregiving create undue stress and lowers health outcomes for children and adults alike. To capture the total burden of sepsis, both during inpatient hospitalization and in follow-up, the economic impact, including direct costs and time costs, must be considered. Measurement of spillover effects allows researchers to evaluate and compare the efficiency of interventions, which will become more important as we move to implement programs to limit long-term sequelae. Additionally, characterizing the burden of on family members will allow for the design of interventions targeted toward the caregiver. Through quantitative and qualitative methods, we can more readily understand the current economic issues of pediatric sepsis survivorship, inform policy to mitigate spillover effects, and comprehensively evaluate the impact of longitudinal sepsis interventions.

## Data Availability Statement

The original contributions presented in the study are included in the article/supplementary material, further inquiries can be directed to the corresponding author.

## Author Contributions

EC, HP, and LP contributed to the concept and design of the perspective. EC drafted the initial manuscript and tables. EC, HP, LP, and SW critically evaluated and revised the manuscript. All authors approved the manuscript.

## Funding

EC reports support from grants KL2 TR 002241 and UL1 TR 002240.

## Conflict of Interest

The authors declare that the research was conducted in the absence of any commercial or financial relationships that could be construed as a potential conflict of interest.

## Publisher's Note

All claims expressed in this article are solely those of the authors and do not necessarily represent those of their affiliated organizations, or those of the publisher, the editors and the reviewers. Any product that may be evaluated in this article, or claim that may be made by its manufacturer, is not guaranteed or endorsed by the publisher.
